# Numerical Investigation into the Flow Characteristics of Gas Mixtures in Knudsen Pump with Variable Soft Sphere Model

**DOI:** 10.3390/mi11090784

**Published:** 2020-08-19

**Authors:** Chunlin Du, Xiaowei Wang, Feng Han, Xiaoyu Ren, Zhijun Zhang

**Affiliations:** 1 Beijing Institute of Spacecraft Environment Engineering, Beijing 100094, China; to_cl2004@126.com (C.D.); 33864949@163.com (X.R.); 2 School of Mechanical Engineering and Automation, Northeastern University, Shenyang 110819, Liaoning, China; hanfeng1993s@163.com

**Keywords:** thermal creep flow, variable soft sphere model, Knudsen pump, DSMC, rarefied gas

## Abstract

In Knudsen pumps with geometric configuration of rectangle, gas flows are induced by temperature gradients along channel walls. In this paper, the direct simulation Monte Carlo (DSMC) method is used to investigate numerically the flow characteristics of H_2_–N_2_ mixtures in the Knudsen pump. The variable soft sphere (VSS) model is applied to depict molecular diffusion in the gas mixtures, and the results obtained are compared with those calculated from a variable hard sphere (VHS) model. It is demonstrated that pressure is crucial to affecting the variation of gas flow pattern, but the gas concentration in H_2_–N_2_ mixtures and the collision model do not change the flow pattern significantly. On the other hand, the velocity of H_2_ is larger than that of N_2_. The velocities of H_2_ and N_2_ increase if the concentration of H_2_ rises in the gas mixtures. The results of velocity and mass flow rate obtained from VSS and VHS models are different. Finally, a linear relation between the decrease of mass flow rate and the increase of H_2_ concentration is proposed to predict the mass flow rate in H_2_–N_2_ mixtures.

## 1. Introduction

Gas flows induced by temperature gradients are peculiar to rarefied gases and are an interesting research subject in rarefied gas dynamics [1,2,3,4]. Thermal creep flow is a well-known phenomenon which occurs due to tangential temperature gradients along the channel walls, making gases flow from a low temperature side to a high temperature side without any initial pressure gradient. More than 100 years ago, a vacuum pump based on the thermal creep effect was first proposed by Knudsen Martin, and was then called the Knudsen pump [5]. For a long time since the seminal work of Knudsen, the Knudsen pump had not been widely applied because thermal creep flow is generated in a rarefied gas.

In recent years, with the progress of micro-fabrication techniques and the advent of micro-electromechanical systems (MEMS), researchers have become increasingly interested in thermal creep flow and the Knudsen pump [6,7,8,9,10,11,12,13,14]. Wang et al. [15] have recently reviewed more than 200 works in the literature to present a comprehensive literature review on the origin of Knudsen pump and its historical development. Based on the design of Knudsen pump, researchers have suggested some configuration variants [16,17,18,19,20,21,22,23,24]. It demonstrates that rectangular layout returns the highest performance, while the performance of straight-curved geometry is the worst [18]. The reason for this is that the linear nature of a rectangular Knudsen pump does not require a gross change in mean flow direction [18]. For Knudsen pumps in rectangular layouts, in practice [12,13,25,26,27,28,29], with the help of micro-machining technologies, heaters are placed on one side of the narrow channels. On the other side of the narrow channels, however, the temperature is decreased by placing a heat sink or the natural convection of the ambient air. As a result, temperature gradients appear along the channel walls, making the gas flow from the cold end to the hot end of the channels. Furthermore, the temperature gradients can be adjusted by changing input power so as to control the gas flow rate [12,13]. In order to observe thermal creep flow phenomena in the numerical simulations, the two extremities of the channel are assumed as isothermal walls at different temperatures, and the temperatures of the channel walls are linearly distributed [16,18,30].

Moreover, with the advancement of computational techniques and the increase of computational speed, Knudsen pump variants with complex structures can be simulated by the method of direct simulation Monte Carlo (DSMC). It is well-known that in order to accurately simulate the molecular collision process to obtain a reliable macroscopic quantity, the use of a more physically realistic collision model is crucial. In previous research on the Knudsen pump, the hard sphere (HS) model [19] and variable hard sphere (VHS) model [20,21,22,23,24,31] are two common molecular models for particle simulations. In fact, the HS model has disadvantages of constant collision cross-section and unrealistic scattering law [32]. Thus, the HS model is not physically realistic or recommended. VHS model [33], however, is the most widely-used molecular model, which is enough for the analysis of rarefied gas flow of single gas. However, in practice, the VHS model is more often used for gas mixtures compared to single gases. Note that in the VHS model, the ratio of diffusion collision cross-section $\sigma_{D}$ to viscosity cross-section $\sigma_{\mu }$ is constantly 1.5, which results in a large deviation from practice when multi-component gas mixtures with diffusion playing an essential role are considered [32,34]. In contrast, the problem can be effectively avoided by applying variable soft sphere (VSS) model [35,36]. Recent research shows [37]: (i) in a high temperature region, molecular models (VSS and VHS) make little difference to simulation results with the temperature ratio, heat transfer ratio, and pressure ratio very close to 1; and (ii) in a low temperature region, the result differences due to molecular models are big, and the heat transfer ratio of the VHS model to VSS model is maximally about 4.3. Thus, the application of the VSS model can reflect the flow characteristics of gas mixtures in a Knudsen pump more realistically, which is important for the development of Knudsen pump and its application to MEMS.

Considering the background mentioned above, the DSMC method and VSS model are used in this paper to discuss the flow characteristics of two-component gas mixtures for different pressures and different gas concentration ratios in a Knudsen pump with rectangular layout. Moreover, the results obtained from VHS and VSS models are compared. The remainder of this paper is structured as follows. In Section 2, configurations of the Knudsen pump are depicted in detail. Collision models, boundary conditions and the corresponding modeling assumptions are briefly presented in Section 3. Simulation results of flow field, thermal field and mass flow rate in the Knudsen pump are given in Section 4. Finally, some conclusions and suggestions are provided in Section 5.

## 2. Problem Statement

**Figure 1:**
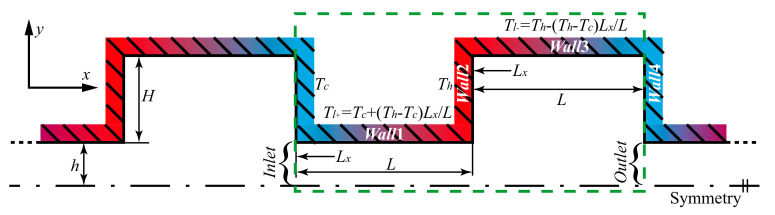
Configuration and geometric parameter.

The studied configuration is composed of a series of alternately connected narrow and wide channels in serial cascades [18,31]. It is up-down symmetrical with a black dot-dashed line, thus only the upper half of the configuration is presented, as shown in Figure 1. The lengths of the narrow and wide channels are equal, *L* = 2 μm. The width of the narrow channel is 2*h* = 1 μm, and that of the wide channel is 2(*h* + *H*) = 3 μm. The walls of two ends of the channels are isothermal with low temperature $T_{c}\;=\;225\;K$ and high temperature $T_{h}=375\;K$ [19,24,31], respectively. Here, the high temperature walls are indicated by *Wall*2, the low temperature walls are *Wall*4, the narrow channel walls are *Wall*1, and the wide channel walls are *Wall*3. On the other hand, the temperature variations between $T_{c}$ and $T_{h}$ are linear on *Wall*1 and *Wall*3 with a temperature gradient of $(T_{h}\;-\;T_{c})/L$. More precisely, the temperature of *Wall*1, $T_{l+}$, increases from $T_{c}$ to $T_{h}$ in the x direction, $T_{l+}\;=T_{c}\;+\;(T_{h}\;-\;T_{c})L_{x}/L$. In contrast, the temperature of *Wall*3, $T_{l-}$, restores from $T_{h}$ to $T_{c}$ in the x direction, $T_{l-}\;=T_{h}\;-\;(T_{h}\;-\;T_{c})L_{x}/L$. $L_{x}$ is the distance of one point on the channel wall from the left end.

The configuration has a periodic pattern with a length 2*L* in the x direction, thus only a length 2*L* of the channel is simulated in the present work. A two-dimensional (2D) simulation domain is indicated by a green dotted box, as shown in Figure 1. The location on the left side where gases flow in is *Inlet*, and the location on the right side where gases flow out is *Outlet*. Information of the simulation domain and other relevant parameters are shown in Section 3.2. Note that since two-dimensional simulations are implemented in this work, a width of 20 μm in the z direction is considered when the mass flow rates are calculated [19,24,31].

## 3. Theoretical Models and Numerical Method

### 3.1. Hard Sphere, Variable Hard Sphere and Variable Soft Sphere Model

**Figure 2:**
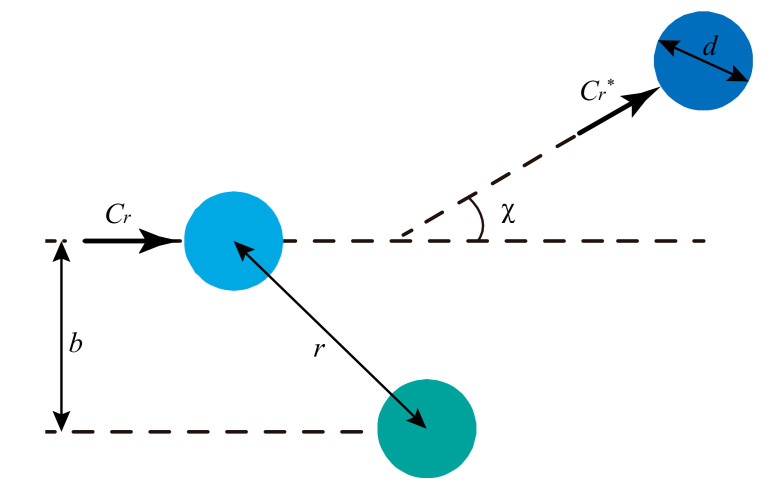
Binary scattering diagram in the fixed scattering center frame of reference.

Two gas molecules with a relative velocity $C_{r}$ and separated by a distance r, approach and collide with each other. After the collision, the two molecules are scattered by an angle χ, with a post-collision relative velocity $C_{r}^{*}$, as shown in Figure 2. In the process of collision, the relative velocities are unchanged, $C_{r}\;=\;C_{r}^{*}$. Additionally, an impact parameter, b, is the separation distance between two molecules measured perpendicular to the relative collision velocity. A head-on collision occurs with b = 0, and no collision happens as b → ∞.

The HS model is the first and simplest molecular interaction model to be used in the simulation of rarefied gas flows [38,39]. Its deflection angle χ and the total collision cross-section $\sigma_{T}$ are [40]:

(1) $$\chi \;=\;2\cos\left(\frac{b}{d_{12}}\right),$$

(2) $$\sigma_{T}\;=\;\pi d_{12}^{2},$$

where $d_{12}\;=\;(d_{1}\;+\;d_{2})/2$ is the distance between the centers of two collision molecules. $d_{1}$ and $d_{2}$ are diameters of these two molecules. If the two collision molecules are the same, then $d_{12}\;=\;d_{1}\;=\;d_{2}$. Regarding the gas molecule of HS model, its diameter $d_{HS}$ can be represented by [40]:

(3) $$d_{HS}\;=\;{\left(\frac{5{\left(mkT_{ref}/\pi \right)}^{1/2}}{16\mu_{ref}}\right)}^{1/2},$$

where m is the molecular mass. $k\;=\;1.38\;\times \;10^{-23}\;J/K$ is a Boltzmann constant. $T_{ref}$ is the reference temperature, and the corresponding gas viscosity is $\mu_{ref}$. It can be seen that the deflection angle and total collision cross-section are constant in HS model, which is independent of the relative translational energy $E_{t}$. In fact, the total collision cross-section decreases with $E_{t}$ increasing, that is, it decreases with the increase of $C_{r}$. Therefore, Bird proposed the VHS model [33].

The VHS model has the same representations of the deflection angle χ and the total collision cross-section $\sigma_{T}$ as the HS model; refer to Equations (1) and (2). However, the molecular diameter of the VHS model $d_{VHS}$ is directly proportional to a function of a certain inverse power of $E_{t}$ [40],

(4) $$d_{VHS}\;=\;{\left(\frac{5{\left(m/\pi \right)}^{1/2}{\left(kT_{ref}\right)}^{\omega }}{8\Gamma (9/2-\omega )\mu_{ref}E_{t}^{\omega -0.5}}\right)}^{1/2}.$$

where $E_{t}\;=\;m_{r}C_{r}^{2}/2$, $m_{r}$ is the collision-reduced mass, $m_{r}\;=\;m_{1}m_{2}/(m_{1}\;+\;m_{2})$. Γ denotes the Gamma function. ω (0.5 ≤ ω ≤ 1.0) is the temperature index of viscosity, depending on the interaction pair. In particular, the equation corresponds to the HS model with ω = 0.5. Although the problem that the total collision cross-section is dependent on relative translational energy is successfully resolved by applying VHS model, both VHS and HS models have the same representation for the ratio of viscosity cross-section $\sigma_{\mu }$ to diffusion cross-section $\sigma_{D}$, and the value is constant [40],

(5) $$\frac{\sigma_{\mu }}{\sigma_{D}}\;=\;\frac{2}{3}.$$

As mentioned above, that shortcoming of the VHS model leads to diffusion coefficients in a poor agreement with the practical measurement results [35,36,40]. Thus Koura and Matsumoto put forward the VSS model [35,36].

The VSS model has the same equation for total collision cross-section as the VHS model, thus Equation (2) is needed. However, the VSS model includes an additional model parameter α to control the scattering angle. The scattering angle is [40]:

(6) $$\chi \;=\;2\cos{\left(\frac{b}{d_{12}}\right)}^{1/\alpha }.$$

Usually, α ranges between 1 and 2. Note that Equation (6) is transformed into Equation (1) at α = 1. Viscosity cross-section and diffusion cross-section of the VSS model are respectively as follows [40],

(7) $$\sigma_{\mu }\;=\;\frac{2\pi S_{\mu }d_{12}^{2}}{3},$$

(8) $$\sigma_{D}\;=\;\pi S_{D}d_{12}^{2},$$

where $S_{\mu }\;=\;6\alpha /\left((\alpha \;+\;1)(\alpha \;+\;2\right))$ is a soft coefficient of viscosity cross-section, while $S_{D}\;=\;2/\left(\alpha \;+\;1\right)$ is a soft coefficient of diffusion cross-section. It can be seen that the ratio of viscosity cross-section to diffusion cross-section is a variable related to α,

(9) $$\frac{\sigma_{\mu }}{\sigma_{D}}\;=\;\frac{2\alpha }{\alpha +2}.$$

By choosing the parameter α, the actual viscosity and diffusion cross-sections for a given interaction potential can be generated. In particular, the relation between viscosity and diffusion cross-sections is obtained with α = 1. In the VSS model, the gas molecular diameter $d_{VSS}$ is [40]:

(10) $$d_{VSS}\;=\;{\left(\frac{5(\alpha \;+\;1)(\alpha \;+\;2){\left(m/\pi \right)}^{1/2}{\left(kT_{ref}\right)}^{\omega }}{16\alpha \Gamma (9/2-\omega )\mu_{ref}E_{t}^{\omega -0.5}}\right)}^{1/2}.$$

Equation (10) can be transformed into Equation (4) for the gas molecular diameter of the VHS model if α = 1. And if α = 1 and ω = 0.5, the representation of the HS model is obtained, see Equation (3). Moreover, for the HS, VHS, and VSS models, the post-collision relative velocities of molecules can be represented by [40]:

(11) $$C_{r(HS)}^{*}\;=\;C_{r(VHS)}^{*}=\left\{\begin{array}{l} u_{r}^{*}=\cos\chi \sqrt{u_{r}^{2}+v_{r}^{2}+w_{r}^{2}}\\ v_{r}^{*}=\sin\chi \cos\phi \sqrt{u_{r}^{2}+v_{r}^{2}+w_{r}^{2}}\\ w_{r}^{*}=\sin\chi \sin\phi \sqrt{u_{r}^{2}+v_{r}^{2}+w_{r}^{2}} \end{array}\right.,$$

(12) $$C_{r(VSS)}^{*}=\left\{\begin{array}{l} u_{r}^{*}=\cos\chi u_{r}+\sin\chi \sin\phi \sqrt{v_{r}^{2}+w_{r}^{2}}\\ v_{r}^{*}=\cos\chi v_{r}+\sin\chi \left(c_{r}w_{r}\cos\phi -u_{r}v_{r}\sin\phi \right)/\sqrt{v_{r}^{2}+w_{r}^{2}}\\ w_{r}^{*}=\cos\chi w_{r}-\sin\chi \left(c_{r}v_{r}\cos\phi +u_{r}w_{r}\sin\phi \right)/\sqrt{v_{r}^{2}+w_{r}^{2}} \end{array}\right..$$

where $\cos\chi \;=\;2R_{F}^{1/\alpha }\;-\;1,\;\;\phi \;=\;2\pi R_{F}.$$R_{F}$ is a random number uniformly distributing between 0 and 1. $u_{r}$, $v_{r}$, and $w_{r}$ are components of pre-collision relative velocity $C_{r}$ in the x, y, and z directions, respectively. Again, $u_{r}^{*}$, $v_{r}^{*}$, and $w_{r}^{*}$ are components of post-collision relative velocity $C_{r}^{*}$ in the x, y, and z directions, respectively. It can be seen that the differences of the molecular collisions among the HS, VHS and VSS models are significant. Note that VSS model can not only reflect the molecular diffusion effect well, but also has almost the same computational simplicity as the VHS model [32]. Thus, applying the VSS model can illustrate the gas movement rules better for gas mixtures, so as to improve the accuracy of the simulation results.

### 3.2. Boundary Condition

As depicted above, the computational domain is the channels surrounded by a green dotted box of Figure 1. *Wall*1, *Wall*2, *Wall*3, and *Wall*4 are all set as wall boundary conditions. Temperatures increasing and decreasing from left to right are, respectively, imposed on *Wall*1 and *Wall*3. Regarding *Wall*2 and *Wall*4, the temperatures remain isothermal and are $T_{h}$ and $T_{c}$, respectively. Since channels within only one periodic length are simulated, periodic boundary conditions are implemented at the *Inlet* and *Outlet*. Moreover, only the upper half of the channels is simulated, thus the symmetry plane is set as a symmetrical boundary condition.

### 3.3. Direct Simulation Monte Carlo Method and Solver

For the continuum regime, the familiar Navier–Stokes (NS) continuum-fluid equations are totally applicable. With the increase of gas rarefaction degree, velocity slip and temperature jump are observed at a surface. The range of validity of the NS equations can be extended to the slip regime by applying velocity-slip and temperature-jump boundary conditions. However, once entering the transition and free-molecular regimes, the NS equations are no longer applicable, and instead the Boltzmann equation must be used to describe the flow behaviors accurately. The DSMC method, first proposed by Bird [39,41], is a stochastic atomistic technique for numerical simulation of rarefied gas flows [40,42]. The DSMC method does not directly solve the Boltzmann equation, but simulates the real physics that the Boltzmann equation represents. It is shown that the DSMC method can be highly effective [43,44,45] at solving the Boltzmann equation. In addition, this method has already been widely used in the research of Knudsen pumps [19,20,21,22,23,24,31] and other rarefied gas flows induced by temperature fields [1,2,37,45].

The DSMC method uses a large number of representative simulation particles to represent the real gas behaviors, so as to simulate the rarefied gas flows. The information of the locations, velocities and energy of the simulation particles are saved in the computers. Within each time step, the information of the simulation particles changes with the molecular movement, molecular collision, and interaction with the boundary walls. The microscopic quantities of the particles in each cell of the spatial grid are sampled and then averaged to calculate the corresponding macroscopic flow properties.

In this work, an open source DSMC solver, dsmcFoamPlus [46], is applied to simulate the flow structure in a Knudsen pump. In all simulations considered, the gas is binary mixtures of H_2_–N_2_. VHS and VSS models are applied to particle collision scattering. The Larsen–Borgnakke (LB) model [47] is used to achieve the energy exchange of translational energy and internal energy in the process of the molecular collisions. Because the internal temperature of the Knudsen pump is lower than the corresponding species characteristic vibrational temperature, the contribution of the vibrational energy is neglected. No-time counter (NTC) scheme [40] for collision frequency calculation is employed to select collision pairs. Moreover, for all gas-surface interaction phenomena on the walls of the simulated configuration, the well-known Maxwell diffuse reflection model is adopted. The simulation domain is discretized by a uniform grid with a side-length of $\Delta x\;=\;\Delta y\;<\;\lambda_{min}/3$ of one computational cell [31]. $\lambda_{min}$ is a minimum chosen from the mean free paths of H_2_ and N_2_, $\lambda_{min}\;=\;\min\;(\lambda_{H_{2}},\lambda_{N_{2}})$. The time step Δt for the simulation is considered with $\Delta t\;<\;\Delta x/c_{\max}$. $c_{\max}$ is a maximum chosen from the characteristic velocities of H_2_ and N_2_, $c_{\max}\;=\;\max\;(c_{H_{2}},c_{N_{2}})$. The number of gas particles represented by a single DSMC particle is adjusted, and to avoid statistical noises, at least 20 particles are set in each cell initially.

## 4. Results and Discussion

The influence of pressures (71.495, 14.3, and 1.43 kPa) [31] and the concentration ratios of H_2_ to N_2_ (1:1, 4:1 and 1:4) in H_2_–N_2_ mixtures on gas flow characteristics is investigated. In addition, a comparison between the results of VSS and VHS models is given. Knudsen numbers (Kn = λ/(2h)) obtained from the VHS model for different pressures are listed in Table 1. Note that the mean free path of N_2_ $\lambda_{N_{2}}$ is smaller under the same conditions, thus, $\lambda \;=\;\lambda_{N_{2}}$.

### 4.1. Temperature Distribution and Flow Characteristics

**Figure 3:**
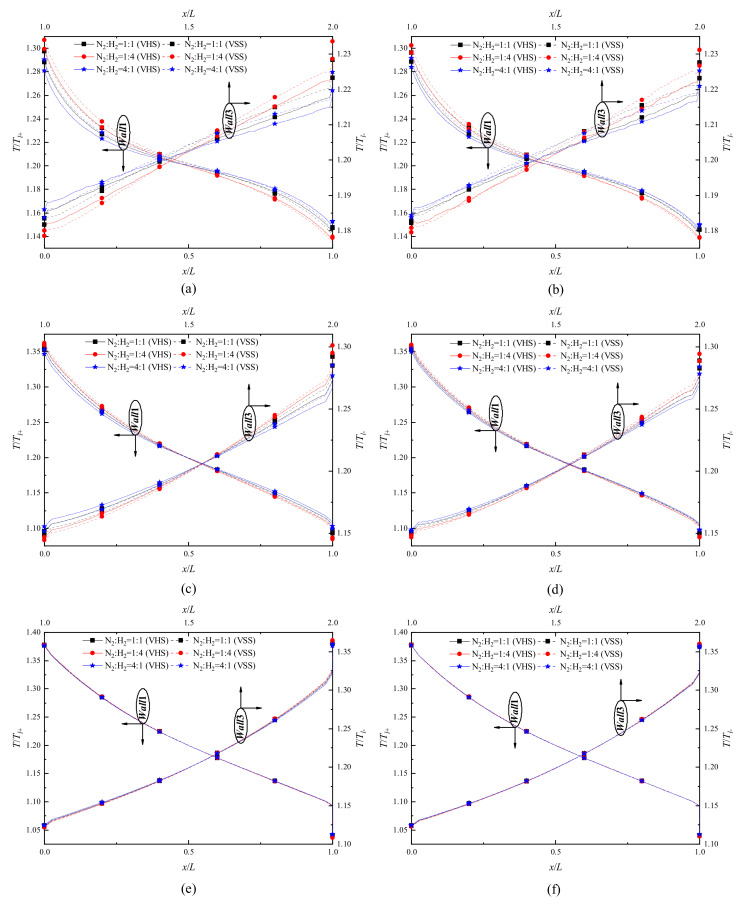
Temperature jump distributions of H_2_ and N_2_ on the *Wall*1 and *Wall*3 surfaces for different pressures. (**a**) P = 71.495 kPa, gas species: H_2_, (**b**) P = 71.495 kPa, gas species: N_2_, (**c**) P = 14.3 kPa, gas species: H_2_, (**d**) P = 14.3 kPa, gas species: N_2_, (**e**) P = 1.43 kPa, gas species: H_2_, (**f**) P = 1.43 kPa, gas species: N_2_.

Figure 3 presents temperature jump distributions of H_2_ and N_2_ on *Wall1* and *Wall3* surfaces for different pressures. In each of the sub-figures, the influence of different H_2_ concentration values is also illustrated. For all pressures considered, the intensity of gas temperature jump on *Wall*1 surfaces weakens, while that on *Wall*3 surfaces strengthens, with the increase of x. The former corresponds to a temperature elevation, but the latter corresponds to a temperature reduction.

The curves at P = 71.495 kPa show that the gas temperature jumps on *Wall*1 surfaces are non-linearly dependent on x, while the dependence of the gas temperature jumps on *Wall*3 surfaces on x is almost linear, as shown in Figure 3a,b. That is likely because the size of narrow channels is smaller than that of wide channels, leading to a stronger rarefied gas effect (non-equilibrium effects) in the narrow channels. More precisely, in the narrow channels, the stronger non-equilibrium effects make the ratio of gas temperature on the surface to the temperature of the surface itself change non-linearly, but the variation of the ratio is linear in the wide channels. For all pressures, the difference value between $T/T_{l+}$ and $T/T_{l-}$ is not big, but the value range of $T/T_{l+}$ is obviously larger. With the pressure decreasing, the degree of gas rarefaction in the wide channels strengthens (Kn increases), and the dependence of the gas temperature jump on *Wall*3 surfaces on x is changed from linear to non-linear, as shown in Figure 3c–f. Similarly, it can be found that with a decrease of pressure, the curves of gas temperature jump on *Wall*1 surfaces are getting smoother; in contrast, the curves of gas temperature jump on *Wall*3 surfaces become steeper. On the other hand, the existence of a sharp edge can lead to a dramatic variation of the gas temperature near it [19,23,24], thus, the temperature jump curves on *Wall*1 and *Wall*3 change dramatically at the extremities. The phenomenon becomes significant when the pressures decreases, as shown in Figure 3.

By comparing the gas temperature jumps based on the VHS and VSS models, it can be found that: (i) when the pressure is large, the VHS model returns a relatively low result in a low temperature region, but in a high temperature region, it returns a relatively high value, as shown in Figure 3a–d; (ii) the VHS and VSS models return almost the same results with small pressures, as shown in Figure 3e,f. That is, with a large pressure, the simulation results will further deviate from the real situations if the VHS model is used. The gas temperature jumps obtained from the VHS and VSS models are different, but the difference of H_2_ on *Wall*1 and *Wall*3 surfaces are obviously larger than that of N_2_, as shown in Figure 3a–d. It means that compared to heavyweight gases, the transport of lightweight gases is more strongly affected by molecular collision models. On the other hand, the influence of H_2_ concentration is illustrated in Figure 3a–d. The curves of temperature jumps of H_2_ and N_2_ on *Wall*1 and *Wall*3 surfaces are getting steeper with H_2_ concentration increasing, that is, the slopes (absolute value) of the curves increase. The phenomenon likely results from an enhancement of the degree of gas rarefaction (Kn increases) in the flow field. More precisely, Kn is a function of pressure, temperature, and gas species (diameter). Thus, the overall diameter of gas mixtures decreases when increasing the concentration of H_2_ in H_2_–N_2_ mixtures, which leads to an increase of Kn in the channels.

**Figure 4:**
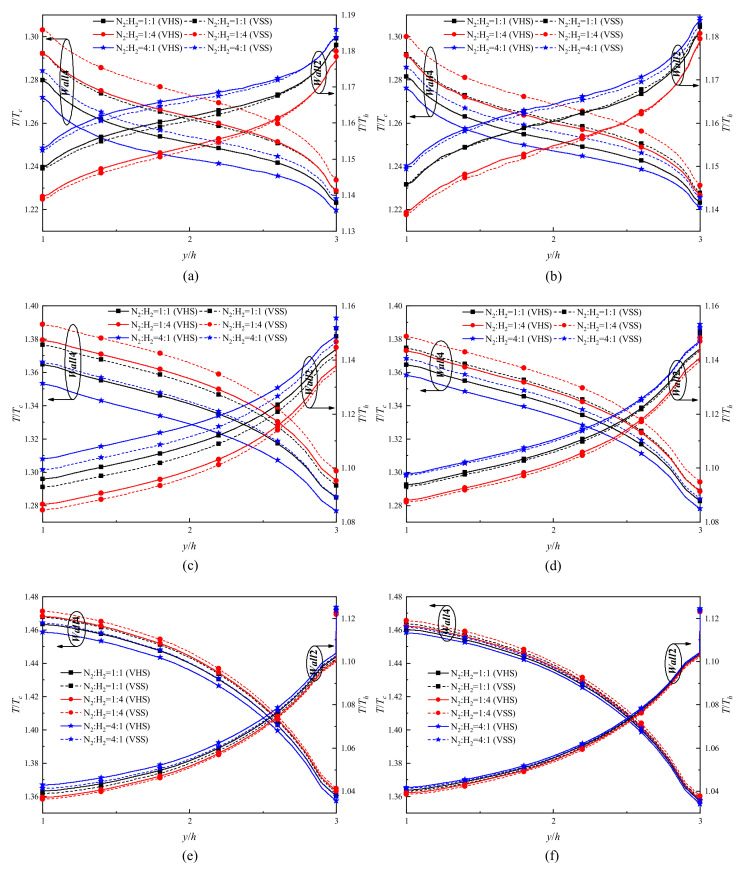
Temperature jump distributions of H_2_ and N_2_ on the *Wall*2 and *Wall*4 surfaces for different pressures. (**a**) P = 71.495 kPa, gas species: H_2_, (**b**) P = 71.495 kPa, gas species: N_2_, (**c**) P = 14.3 kPa, gas species: H_2_, (**d**) P = 14.3 kPa, gas species: N_2_, (**e**) P = 1.43 kPa, gas species: H_2_, (**f**) P = 1.43 kPa, gas species: N_2_.

Turning to the gas temperature jumps on *Wall*2 and *Wall*4 surfaces, phenomena similar to that presented above can be found, as shown in Figure 4. For all pressures discussed, with y increasing, the intensity of gas temperature jumps on *Wall*2 surfaces strengthens, while that on *Wall*4 surfaces weakens. Note that gas temperature jumps are more significant in a low temperature region (*Wall*4 surface) than in a high temperature region (*Wall*2 surface). Moreover, with the pressure decreasing, the intensity of gas temperature jumps on *Wall*2 surfaces weakens, while that on *Wall*4 surfaces strengthens. Also, this phenomenon appears when the concentration of H_2_ increases in H_2_–N_2_ mixtures. It is assumed that the phenomenon results from an enhancement of gas rarefaction degree in the channels due to the reduction in pressure or gas diameter. More precisely, as the gas rarefaction degree rises, the heat transfer of *Wall*2 and *Wall*4 surfaces is decreased, which leads to a decrease of gas temperature on *Wall*2 hot surfaces (the temperature jump intensity weakens), in contrast, the gas temperatures on *Wall*4 cold surfaces increase (the temperature jump intensity strengthens). These rules can be observed on *Wall*1 and *Wall*3 surfaces, as shown in Figure 3a–d. Therefore, the phenomena of the increase of curve slope with the increase of rarefaction degree on *Wall*1 and *Wall*3 surfaces appear, as stated in the above paragraph.

On the other hand, by comparing the temperature jumps calculated from the VHS and VSS models, it can be found that: (i) the VHS model returns a relatively low result in a low temperature region, but returns a relatively high result in a high temperature region; (ii) the results returned by the VHS model is closer and closer to the results returned from the VSS model with the pressure decreasing, which are also observed on *Wall*1 and *Wall*3 surfaces, as discussed above. Note that the first finding is more significant for H_2_ (lightweight gas), while the second finding is more significant for N_2_ (heavyweight gas).

**Figure 5:**
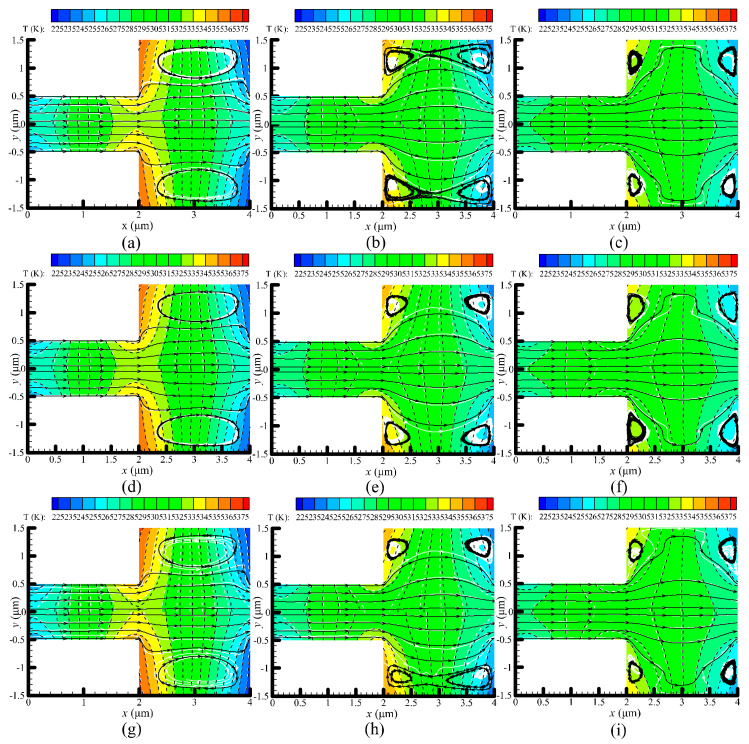
Temperature contours and velocity streamlines for different pressures and gas mixtures (N_2_:H_2_). Dotted black lines: N_2_, while dotted white lines with colored background: H_2_. (**a**) P = 71.495 kPa, N_2_:H_2_ = 1:1; (**b**) P = 14.3 kPa, N_2_:H_2_ = 1:1; (**c**) P = 1.43 kPa, N_2_:H_2_ = 1:1; (**d**) P = 71.495 kPa, N_2_:H_2_ = 1:4; (**e**) P = 1.43 kPa, N_2_:H_2_ = 1:4; (**f**) P = 1.43 kPa, N_2_:H_2_ = 1:4; (**g**) P = 71.495 kPa, N_2_:H_2_ = 4:1; (**h**) P = 14.3 kPa, N_2_:H_2_ = 4:1; (**i**) P = 1.43 kPa, N_2_:H_2_ = 4:1. In each of the sub-figure, the top half (y > 0) shows the results of the variable hard sphere (VHS) model, the bottom half (y < 0) shows the results of the variable hard sphere (VHS) model.

Temperature contours and velocity streamlines predicted by the VHS and VSS models are illustrated in Figure 5, and are compared side-by-side. Overall, the results of the VHS and VSS models are in good agreement. In the VHS model, the temperature contours of N_2_ and H_2_ are almost overlapping, but differences can be observed in the VSS model, which might result from different molecular interactions of these two collision models [40,42]. In particular, the temperature contours of the VHS and VSS models are highly consistent at P = 1.43 kPa, as shown in Figure 5c,f,i. That is because fewer molecular collisions occur in a low pressure environment, which leads to a weak influence of the collision models on the results. Compared to that at P = 1.43 kPa, the difference of temperature contours of the VHS and VSS models exists at P = 71.495 kPa and more significantly, at P = 14.3 kPa. It is presumed that an enhancement of pressure (the increase in molecular collisions) is the reason why the results obtained from the VHS and VSS models are different. In addition, a stronger thermal creep effect at P = 14.3 kPa (refer to the next part) is another factor leading to the obvious difference.

Figure 5 clearly displays that the flow patterns in the narrow channels always remain in a direction from left to right. However, in the wide channels, the flow patterns change significantly for different pressures. For example, a large vortex is generated in the wide channel with P = 71.495 kPa, as shown in Figure 5a,d,g. As the pressure decreases, the large vortex will finally be replaced with two small vortices, as shown in Figure 5c,f,i. The qualitative change rules of the gas flow patterns can also be observed in [31]. No obvious differences of the flow patterns are observed for different collision models (VHS and VSS models) or different concentration ratios of H_2_–N_2_ mixtures. Therefore, it can be concluded that pressure is the crucial factor in changing the gas flow patterns [31], instead of the collision models and carrier gases.

### 4.2. Velocity

**Figure 6:**
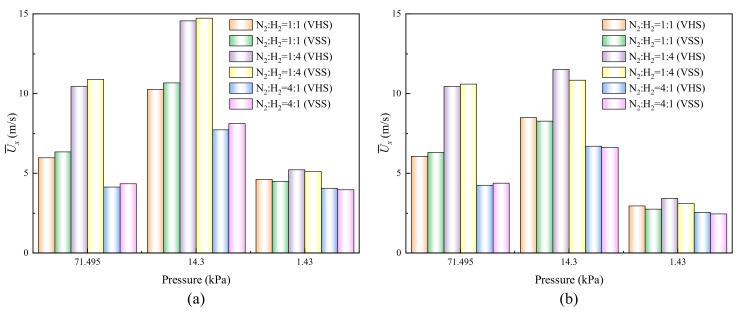
Mean velocities over an *x* = 2 μm vertical line for different pressures. (**a**) Mean velocities of H_2_. (**b**) Mean velocities of N_2_.

In order to analyze the above flow results quantitatively, mean velocities over a vertical line at *x* = 2 μm (the outlet of narrow channel) are depicted in Figure 6. Although the flow patterns obtained from the VHS and VSS models are highly similar, differences of the velocity values are clearly observed in Figure 6. Evidently, the VHS model returns a relatively low velocity at P = 71.495 kPa, but returns a relatively high velocity at P = 1.43 kPa. The reason is probably that the molecular diffusion effect of the gas mixtures cannot be effectively presented in the VHS model. Note that at P = 14.3 kPa, the VHS model returns a relatively low velocity of H_2_, but returns a relatively high velocity of N_2_, which means that carrier gases is a factor in changing the returned results for different collision models.

On the other hand, both of the velocities of H_2_ and N_2_ increase with the decrease of pressure until P = 14.3 kPa, and then decrease as the pressure continues to decrease. It means that pressure is an important factor in changing thermal creep flow effect, and the maximum velocity usually appears at the initial stage of the transition regime (0.1≤ Kn ≤0.5) [19,20,23,24,31]. The velocities of H_2_ are larger than that of N_2_ in H_2_–N_2_ mixtures for all pressures discussed. In particular, the phenomenon is more significant for low pressures, such as P = 14.3 and 1.43 kPa. That is because the thermal creep effect is closely related to the weight of the gas molecule [48]: lightweight gases have a stronger thermal creep effect, that is, a larger velocity. Thus, when the concentration of H_2_ rises in the gas mixtures, the thermal creep effect is enhanced. This not only leads to an increase in the velocity of H_2_ itself, but also contributes to an increase in the velocity of N_2_. The lightweight gases can promote the movement of the heavyweight gases [31]. Note that although the qualitative change rules of velocity can also be observed in [31], the mean velocity values here are obviously higher. The reason is that H_2_–N_2_ mixtures are considered in this paper, while N_2_-O_2_ mixtures are studied in [31].

### 4.3. Species Separation

**Figure 7:**
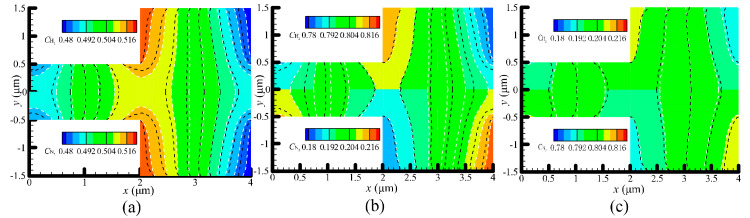
Concentration contours of H_2_ and N_2_ at P = 71.495 kPa. (**a**) N_2_:H_2_ = 1:1, (**b**) N_2_:H_2_ = 1:4, (**c**) N_2_:H_2_ = 4:1. Dotted black lines: VSS model, while dotted white lines with colored background: VHS model. In each of the sub-figure, the top half (y > 0) shows the concentration of H_2_, (y < 0) shows the concentration of N_2_.

**Figure 8:**
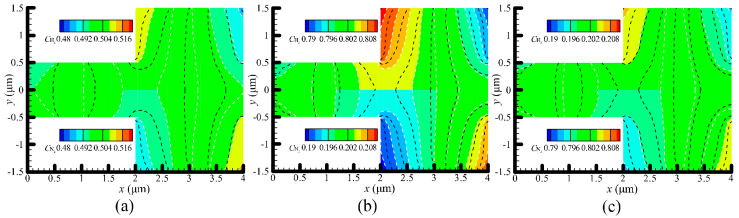
Concentration contours of H_2_ and N_2_ at P = 14.3 kPa. (**a**) N_2_:H_2_ = 1:1, (**b**) N_2_:H_2_ = 1:4, (**c**) N_2_:H_2_ = 4:1. Dotted black lines: VSS model, while dotted white lines with colored background: VHS model. In each of the sub-figure, the top half (y > 0) shows the concentration of H_2_, (y < 0) shows the concentration of N_2_.

Gas species separation can be achieved by the differences between velocities of each gas species in the gas mixtures [22,49,50]. Figure 7 and Figure 8 present the concentration contours of H_2_ and N_2_ at P = 71.495 and 14.3 kPa, respectively. Obviously, lightweight gas (H_2_) has a tendency to accumulate at the hotter regions, while heavyweight gas (N_2_) tends to accumulate at the colder regions in the channel. The similar results are also reported in [22]. As expected, when pressures decrease, the gases accumulating at the hot and cold regions reduce, as shown in Figure 8, which means that majority of gases flowing in from the inlet will flow out of the outlet, with only a small part of them accumulating in the channel. Regarding the concentration contours, the results calculated from the VSS and VHS models are different, as shown in Figure 7 and Figure 8. The differences become more significant when pressures decrease, which likely results from the change of the flow patterns in the channel.

### 4.4. Mass Flow Rate

**Figure 9:**
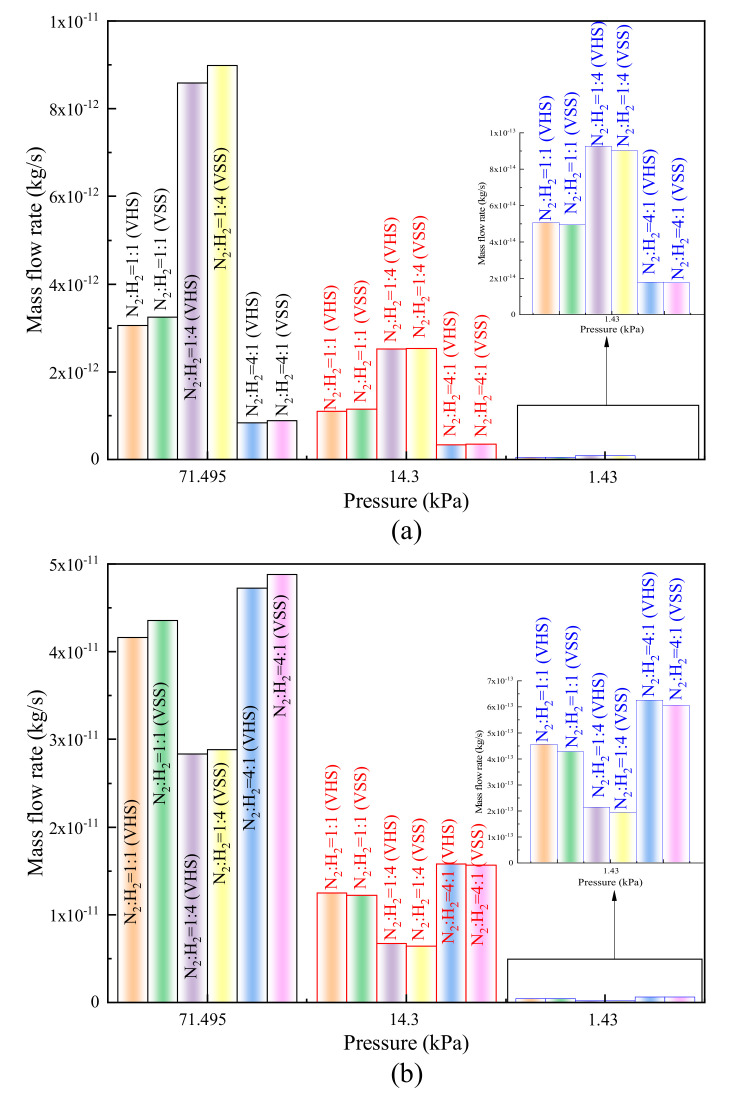
Mass flow rates for different pressures. (**a**) Mass flow rates of H_2_ for different pressures, (**b**) mass flow rates of N_2_ for different pressures.

Figure 9 depicts variations of the mass flow rate for different pressures. Although the velocities show a variation rule increasing first and then decreasing with a decrease in pressure, mass flow rates decrease gradually as pressures decrease. This is mainly because the enhancement of the gas rarefaction degree causes a decrease in the number of molecules. The similar results are also reported in [31]. Furthermore, compared to the values of H_2_, the mass flow rates of N_2_ are obviously higher, which can be expected because N_2_ is heavier than H_2_. On the other hand, the change rules of mass flow rate and velocity caused by different collision models are similar since the mass flow rate is proportional to the velocity.

**Figure 10:**
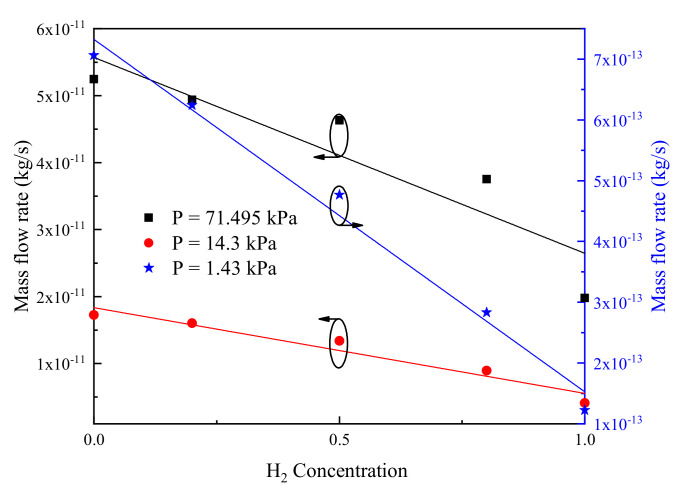
Mass flow rate versus H_2_ concentration, VSS model.

A dimensionless relation which can predict the total mass flow rate based on ambient pressure and concentration of H_2_ is put forward in practice. From Figure 10, it can be found that the total mass flow rates decrease with the concentration of H_2_ increasing, thus that for H_2_–N_2_ mixtures can be represented by:

(13) $${\dot{M}}_{mix}\;=\;{\dot{M}}_{N_{2}}\;+\;SC_{H_{2}}.$$

where, ${\dot{M}}_{N_{2}}$ indicates the mass flow rate in a pure N_2_ system with the same total pressure and temperature as the gas mixtures considered. $C_{H_{2}}$ is the concentration of H_2_, and S is the slope of each line in Figure 10. Thus, the mass flow rate for H_2_–N_2_ mixtures at the same pressure can be predicted if ${\dot{M}}_{N_{2}}$ at a given pressure and S are confirmed. Note that at different operating pressures, the coefficients of determination of the linear fitting (R^2^ coefficient) are 0.84131 (P = 71.495 kPa), 0.94811 (P = 14.3 kPa), and 0.98687 (P = 1.43 kPa). Obviously, the coefficient is low for P = 71.495 kPa, that is, the linear fitting between mass flow rate and H_2_ concentration is not very good. However, with the decrease of pressure, the coefficients obviously rise and become closer to 1.0, which means that the higher the gas rarefaction degree, the better the linear relation between mass flow rate and H_2_ concentration.

## 5. Concluding Remarks

The flow characteristics of H_2_–N_2_ mixtures in the rectangular Knudsen pump were studied by using the DSMC method. The influences of different concentration ratios of H_2_–N_2_ mixtures and different pressures on the gas flow characteristics were investigated well. Moreover, in order to depict the molecular diffusion in the gas mixtures, the VSS model was used for the extension of the previous work [31]. The simulation results obtained from the VSS and VHS models were compared.

The research results show that the changes of gas concentration in H_2_–N_2_ mixtures and collision model do not lead to a significant variation of the flow pattern. In fact, pressure is the crucial factor in changing flow patterns. By comparing the gas temperature jump distributions on the wall surfaces, it can be found that the VHS model returns a relatively low result at low temperature regions, but returns a relatively high result at high temperature regions. In particular, results calculated from the VHS and VSS models are more consistent with each other when pressures decrease. However, regarding the temperature contours in the channels for all pressures considered, the difference of the results calculated from the VHS and VSS models is the largest at P = 14.3 kPa, and that follows at P = 71.495 kPa. This means that the difference of the results returned from VHS and VSS models depends on the strength of thermal creep effect and pressure.

On the other hand, with the pressure decreasing, both the velocities of H_2_ and N_2_ display a change rule increasing first and then decreasing [31]. For all pressures discussed, H_2_ has a larger velocity than N_2_ in H_2_–N_2_ mixtures. When the concentration of H_2_ rises in the gas mixtures, this not only causes an increase in the velocity of H_2_ itself, but also contributes to an increase in the velocity of N_2_. Also, for the velocity and mass flow rate, the results calculated from the VSS model and the VHS model are different. The change rules of mass flow rate and velocity caused by different collision models are similar since the mass flow rate is proportional to the velocity. As expected, the mass flow rate of N_2_ is obviously higher than that of H_2_ due to a heavier gas molecule of N_2_. Moreover, both the mass flow rates of H_2_ and N_2_ decrease gradually with a decrease in pressure. Finally, the mass flow rate linearly relies on the concentration of H_2_ is proposed to predict the mass flow rate for H_2_–N_2_ mixtures.

## Figures and Tables

**Table 1:** Knudsen numbers for different pressures, VHS model.

Parameter	Value		
Pressure P (kPa)	71.495	14.3	1.43
Kn	0.0767	0.383	3.83

## References

[1] Hassanvand A., Gerdroodbary M.B., Moradi R., Amini Y. (2018). Application of Knudsen thermal force for detection of inert gases. Results Phys..

[2] Vo D.D., Moradi R., Gerdroodbary M.B., Ganji D.D. (2019). Measurement of low-pressure Knudsen force with deflection approximation for gas detection. Results Phys..

[3] Wang X., Zhang Z., Zhang W., Su T., Zhang S. (2020). Impact of Improved Design on Knudsen Force for Micro Gas Sensor. Micromachines.

[4] Zheng Y., Manh T.D., Nam N.D., Gerdroodbary M.B., Moradi R., Tlili I. (2020). Optimization of micro Knudsen gas sensor for high precision detection of SO_2_ in natural gas. Results Phys..

[5] Knudsen M. (1909). Eine revision der gleichgewichtsbedingung der gase. Thermische Molekularströmung. Ann. Phys..

[6] Wang K., Zeng P., Ahn J., Ronney P.D. (2013). A self-sustaining thermal transpiration gas pump and SOFC power generation system. Proc. Combust. Inst..

[7] Zeng P., Wang K., Ahn J., Ronney P.D. (2013). Thermal transpiration based pumping and power generation devices. J. Therm. Sci. Technol..

[8] Qin Y., Gianchandani Y.B. (2016). A fully electronic microfabricated gas chromatograph with complementary capacitive detectors for indoor pollutants. Microsyst. Nanoeng..

[9] Van Toan N., Inomata N., Trung N.H., Ono T. (2018). Knudsen pump produced via silicon deep RIE, thermal oxidation, and anodic bonding processes for on-chip vacuum pumping. J. Micromech. Microeng..

[10] Ye J., Yang J., Zheng J., Ding X., Wong I., Li W., Chen C. (2012). Thermal transpiration effect on the mass transfer and flow behaviors of the pressure-driven hydrogen gas flow. Int. J. Hydrogen Energy.

[11] Ye J., Shao J., Hao Z., Salem S., Zhang Y., Wang Y., Li Z. (2019). Characteristics of thermal transpiration effect and the hydrogen flow behaviors in the microchannel with semicircular obstacle. Int. J. Hydrogen Energy.

[12] An S., Gupta N.K., Gianchandani Y.B. (2013). A Si-micromachined 162-stage two-part Knudsen pump for on-chip vacuum. J. Microelectromech. Syst..

[13] An S., Qin Y., Gianchandani Y.B. (2015). A monolithic high-flow Knudsen pump using vertical Al_2_O_3_ channels in SOI. J. Microelectromech. Syst..

[14] Kugimoto K., Hirota Y., Yamauchi T., Yamaguchi H., Niimi T. (2019). Design and demonstration of Knudsen heat pump without moving parts free from electricity. Appl. Energy.

[15] Wang X., Su T., Zhang W., Zhang Z., Zhang S. (2020). Knudsen pumps: A review. Microsyst. Nanoeng..

[16] Bond D.M., Wheatley V., Goldsworthy M. (2014). Numerical investigation of curved channel Knudsen pump performance. Int. J. Heat Mass Transf..

[17] Chen J., Baldas L., Colin S. (2014). Numerical study of thermal creep flow between two ratchet surfaces. Vacuum.

[18] Bond D.M., Wheatley V., Goldsworthy M. (2016). Numerical investigation into the performance of alternative Knudsen pump designs. Int. J. Heat Mass Transf..

[19] Chen J., Stefanov S.K., Baldas L., Colin S. (2016). Analysis of flow induced by temperature fields in ratchet-like microchannels by Direct Simulation Monte Carlo. Int. J. Heat Mass Transf..

[20] Shahabi V., Baier T., Roohi E., Hardt S. (2017). Thermally induced gas flows in ratchet channels with diffuse and specular boundaries. Sci. Rep..

[21] Baier T., Hardt S., Shahabi V., Roohi E. (2017). Knudsen pump inspired by Crookes radiometer with a specular wall. Phys. Rev. Fluids.

[22] Baier T., Steffen H. (2020). Gas separation in a Knudsen pump inspired by a Crookes radiometer. Microfluid. Nanofluid..

[23] Lotfian A., Roohi E. (2019). Radiometric flow in periodically patterned channels: Fluid physics and improved configurations. J. Fluid Mech..

[24] Wang X., Zhang Z., Zhang W., Zhang P., Zhang S. (2019). Numerical simulation of thermal edge flow in ratchet-like periodically patterned micro-channels. Int. J. Heat Mass Transf..

[25] Kugimoto K., Hirota Y., Kizaki Y., Yamaguchi H., Niimi T. (2017). Performance prediction method for a multi-stage Knudsen pump. Phys. Fluids.

[26] Kugimoto K., Hirota Y., Yamauchi T., Yamaguchi H., Niimi T. (2018). A novel heat pump system using a multi-stage Knudsen compressor. Int. J. Heat Mass Transf..

[27] Yamaguchi H., Rojas-Cárdenas M., Perrier P., Graur I., Niimi T. (2014). Thermal transpiration flow through a single rectangular channel. J. Fluid Mech..

[28] Rojas-Cárdenas M., Graur I., Perrier P., Méolans J.G. (2013). Time-dependent experimental analysis of a thermal transpiration rarefied gas flow. Phys. Fluids.

[29] Cardenas M.R., Graur I., Perrier P., Meolans J.G. (2012). An experimental and numerical study of the final zero-flow thermal transpiration stage. J Therm. Sci. Technol..

[30] Quesada G.L., Tatsios G., Valougeorgis D., Rojas-Cárdenas M., Baldas L., Barrot C., Colin S. (2020). Thermally driven pumps and diodes in multistage assemblies consisting of microchannels with converging, diverging and uniform rectangular cross sections. Microfluid. Nanofluid..

[31] Zhang Z., Wang X., Zhao L., Zhang S., Zhao F. (2019). Study of flow characteristics of gas mixtures in a rectangular Knudsen pump. Micromachines.

[32] Prasanth P.S., Kakkassery J.K. (2008). Molecular models for simulation of rarefied gas flows using direct simulation Monte Carlo method. Fluid Dyn. Res..

[33] Bird G.A. (1981). Monte Carlo simulation in an engineering context. Prog. Astronaut. Aeronaut.

[34] Shen C. (2005). Rarefied Gas Dynamics: Fundamentals, Simulations and Micro Flows.

[35] Koura K., Matsumoto H. (1991). Variable soft sphere molecular model for inverse-power-law or Lennard-Jones potential. Phys. Fluids A.

[36] Koura K., Matsumoto H. (1992). Variable soft sphere molecular model for air species. Phys. Fluids A.

[37] Wang X., Zhang W., Su T., Zhang S., Zhang Z. (2020). Numerical investigation into the low-pressure detection sensor performance of hydrogen gas with variable soft sphere molecular model. Int. J. Hydrogen Energy.

[38] Haviland J.K., Lavin M.L. (1962). Application of the Monte Carlo method to heat transfer in a rarefied gas. Phys. Fluids.

[39] Bird G.A. (1963). Approach to translational equilibrium in a rigid sphere gas. Phys. Fluids.

[40] Bird G.A. (1994). Molecular Gas Dynamics and the Direct Simulation Monte Carlo of Gas Flows.

[41] Bird G.A. (1965). Shock-wave structure in a rigid sphere gas. Proceedings of the 4th International Symposium on Rarefied Gas Dynamics.

[42] Bird G.A. (1976). Molecular Gas Dynamics.

[43] Vargas M., Tatsios G., Valougeorgis D., Stefanov S. (2014). Rarefied gas flow in a rectangular enclosure induced by non-isothermal walls. Phys. Fluids.

[44] Balaj M., Roohi E., Akhlaghi H. (2015). Effects of shear work on non-equilibrium heat transfer characteristics of rarefied gas flows through micro/nanochannels. Int. J. Heat Mass Tran..

[45] Balaj M., Roohi E., Akhlaghi H., Myong R.S. (2014). Investigation of convective heat transfer through constant wall heat flux micro/nano channels using DSMC. Int. J. Heat Mass Tran..

[46] White C., Borg M.K., Scanlon T.J., Longshaw S.M., John B., Emerson D.R., Reese J.M. (2018). dsmcFoam+: An OpenFOAM based direct simulation Monte Carlo solver. Comput. Phys. Commun..

[47] Borgnakke C., Larsen P.S. (1975). Statistical collision model for Monte Carlo simulation of polyatomic gas mixture. J. Comput. Phys..

[48] Kosuge S., Takata S. (2008). Database for flows of binary gas mixtures through a plane microchannel. Eur. J. Mech. B Fluids.

[49] Nakaye S., Sugimoto H., Gupta N.K., Gianchandani Y.B. (2015). Thermally enhanced membrane gas separation. Eur. J. Mech. B Fluids.

[50] Nakaye S., Sugimoto H. (2016). Demonstration of a gas separator composed of Knudsen pumps. Vacuum.

